# Evaluation of indoor air quality and its symptoms in office building – A case study of Mashhad, Iran

**DOI:** 10.1016/j.dib.2018.07.051

**Published:** 2018-07-27

**Authors:** Zahra Atarodi, Kamaladdin Karimyan, Vinod Kumar Gupta, Morteza Abbasi, Masoud Moradi

**Affiliations:** a Students Research Committee, Gonabad University of Medical Sciences, Gonabad, Iran; b Environmental Health Research Center, Kurdistan University of Medical Sciences, Sanandaj, Iran; c Department of Environmental Health Engineering, School of Public Health, Tehran University of Medical Sciences, Tehran, Iran; d Department of Applied Chemistry, University of Johannesburg, Johannesburg, South Africa; e Behvarz Training Center, Torbat Heydariyeh University of Medical Sciences, Torbat Heydariyeh, Iran; f Research Center for Environmental Determinants of Health, Kermanshah University of Medical Sciences, Kermanshah, Iran

**Keywords:** Indoor air quality, Sick building syndrome, Air pollution, Mashhad

## Abstract

Air pollution sources in indoor area are one of the main factors for reducing indoor air quality of locations. In the current research, factors affecting indoor air quality and its symptoms are evaluate as case study in an office building in Mashhad (Iran). In order to conduct this descriptive-analytical study, gas pollutants affecting indoor air quality were determined using portable analysis systems. Alberta Indoor Air Quality Toolkit was used in order to study sick building syndrome. Findings indicated that 21% of staffs viewed labor environment conditions as inappropriate and they were mostly compliant about feeling of dusty air, fatigue and headache. In addition, findings showed that O_3_, VOC, PM_10_, PM_2.5_, CO, CO_2_ parameters, Formaldehyde, temperature, sound and humidity were at standard level. Indicators of indoor air quality, in addition to the stress and depression interference on employee performance and satisfaction were at acceptable level.

**Specifications Table**

**Table:** 

Subject area	Environmental health sciences
More specific subject area	Indoor air pollution
Type of data	Table
How data was acquired	In order to do present study, O_3_, VOC, PM, CO, CO_2_ pollutants affecting indoor air quality were determined using portable analysis systems. In addition, the “sick building syndrome” was studied using Alberta Indoor Air Quality Toolkit.
Data format	Raw, analyzed
Experimental factors	The following scoring method was used for investigating frequency of staff ideas on environmental factors of indoor air: highly inappropriate (1–20), inappropriate (21–40), normal (41–60), appropriate (61–80) and highly appropriate (81–100).
Experimental features	Sampling and analysis of pollutants (O_3_, VOC, PM_10_, PM_2.5_, CO and CO_2_) in the collected samples were carried out according to the standard method. Smoke (wind direction) and sound test using sound level meter model CEL450 was used for air flow in 60 stations in the building.
Data source location	Mashhad city, Iran
Data accessibility	Data are included in this article

**Value of the data**

• The quality of indoor air, such as air quality of outside the building, is important for human health [1], [2], [3]. The data of this study is to evaluation of indoor air quality and its symptoms are investigated as case study in an office building in Mashhad (Iran).
• In Iran, most air quality studies have been related to outdoor environments [4], [5], [6], [7], [8], [9]. While, given the fact that employees in offices as well as housewives (more than others) spend their time inside buildings, so for those people, the indoor air quality level may be more important than outdoor [10], [11], [12]. The data emphasizes continuous monitoring of indoor air quality.
• The result revealed that air condition is very important for health and efficiency of residents. So continuous air quality monitoring is required.
• The data showed that O_3_, VOC, PM_10_, PM_2.5_, CO, CO_2_ parameters, as well as formaldehyde, temperature, and humidity were at standard level.
• The obtained data can provide a basis research for future similar studies in relation to investigating of indoor air pollution.

## 1 Data

Table 1 gives average results for indoor air quality indexes in the office building under study. Minimum and maximum measured sizes were related to volatile organic compounds as 538 and 1270 ppb in the first and underground floors, which were in comparison with higher than average volatile organic compounds measured in the balcony section. Concentrations of ozone and formaldehyde contaminants were all so low that they were not detected by the diagnostic method of gas detectors.

**Table 1:** Average indoor air quality indices.

**Indicator**	**Number of stations**	**Minimum measured value**	**Maximum measured value**	**Mean and standard deviation (in total classes)**	**Standard value (free air)**	**Unit**
Carbon dioxide	50	309 (3 floor)	704 (under floor)	456.5 ± 19.2	12 ± 189.85	ppm
Carbon monoxide	35	0 (2 floor)	7 (under floor)	2.0 ± 42.3	1.0 ± 8.2	ppm
Volatile organic compounds (VOCs)	60	538 (1 floor)	1270 (under floor)	534.74 ± 21.29	109 ± 310.7	ppm
Formaldehyde	15	0	0	0	0	ppm
Ozone	15	0	0	0	0	ppm
Total particles (TSP)	10	12.5 (3 floor)	21.5 (2 stair)	16.2 ± 17.59	14.3 ± 2.36	µg/m^3^
Particles less than 10 μm (PM_10_)	10	4.3 (2 floor)	9.7 (3 stair)	6.2 ± 68.16	9.1 ± 3.14	µg/m^3^
Particles less than 2.5 μm (PM_2.5_)	10	2.5 (1 floor)	11.12 (3 stair)	6.0 ± 44.43	6.1 ± 6.09	µg/m^3^
Voice	60	55 (3 floor)	65 (under floor)	58.3 ± 17.18	1 ± 64.43	dB

The highest reported concentration of total particles was 21.5 micrograms per cubic meter in open air in the second floor and the lowest was 12.5 micrograms per cubic meter in open air in the third floor, which was lower, compared to average total particle concentration in open air (14.2 micrograms per cubic meter in open air). The maximum and minimum concentrations of particles less than PM_10_ were 9.7 on the third and fourth floors on the second floor and 3.9 micrograms per cubic meter in open air, respectively. The maximum and minimum values of particles less than PM_2.5_ were 11.12 and 2.5 micrograms per cubic meter in open air, respectively, for floors 3 and 2, which was lower than average concentration of these particles in open air (6.6 micrograms per cubic meter in open air). sound pressure level in all measured points was more than 55 dB, so that the highest sound pressure level was achieved in the underground floor (65 dB) and the lowest level was obtained in the third floor (55 dB). The average open air sound pressure level was also measured at 64 dB. In addition to the instantaneous sound pressure level, the equivalent level was measured at a time of about 10 min, with the highest and lowest values of 64.5 and 56.26 dB, respectively, for the underground and first floors, respectively. Investigation of the direction and mode of air flow in various floors of the building using smoke test showed that in some places (about one third of the cases), the direction or distribution of smoke flow from the valves or slots to the return air vents is not appropriate. According to the measurements carried out in this study, the minimum temperature (15.6 °C) was related to the western third floor and the maximum temperature of 5.3 °C was related to the eastern first floor. The highest relative humidity was observed in the underground floor, 43.6% and the lowest in the third floor as 26.3%.

Table 2 gives health consequences reported by the staff working in the building (%) resulting from investigation of sick building syndrome symptoms. Out of 80 distributed questionnaires, 70 ones with average age of 39.25 years and SD of 7.54 completed the questionnaires. five person (71.4%) were females, 20 person (28.57%) were males. 60 person (85.71%) were married and 10 person (14.28%) were single. 100% of the staffs under study were present in the building over 6 h, 100% of staff entered the building before 8:00 pm and 90% left the building after 15.30. 5 ones (7.1%) were smokers, 3 of whom smoked during work at the building. On the other hand, 60 ones (85.71%) stated that their colleagues smoked during the work, and overall about 27% of staffs viewed building conditions in terms of smoking smell as inappropriate and were complaint about it. results showed that highest complaints were about feeling of dusty air, fatigue and headache.

**Table 2:** Health complications reported by employees in the building (%).

**Row**	**Question**	**Never**	**Rarely**	**Sometimes**	**Always**
1	Headache	17	27	49	7
2	Fever	66	25	9	0
3	Dizziness	37	30	25	8
4	Fatigue	7	26	56	1
5	Drowsiness	15	36	47	2
6	Weakness	30	32	28	10
7	Nausea	78	20	2	0
8	Respiratory problems	60	23	16	1
9	Muscle pain, arm or hand	22	34	37	7
10	Chest pain or chest tightness	47	26	22	5
11	Back pain	31	30	27	12
12	Itching the eyes	22	38	34	6
13	Neck pain	17	36	41	6
14	Problems with vision matching and blurred vision	35	40	20	5
15	Burning or sore throat	65	22	11	2
16	Burning or nasal itching	57	25	12	6
17	Symptoms of cold or flu	28	44	25	3
18	Depression	54	25	19	2
19	Create a problem in focus	22	37	36	5
20	Tense or nervous	21	46	27	6
21	Itching, swelling or dry skin	53	26	17	4
22	Feeling cold in the hands or feet	44	29	19	8
23	Feeling heavy air	17	27	39	17
24	Average	36.73	30.60	26.86	5.78

As observed, overall 27% and 6% of staff under study stated that they experience the mentioned consequences sometimes or always. Table 3 gives frequency of staff ideas on the labor place׳s environmental conditions during working. As observed in this diagram, highest complaints of staff about their work place environmental conditions are related to the sound, ventilation, air flow direction, working space, and situation of working desks toward the windows and colleagues.

**Table 3:** Staff opinions about the environmental factors inside the building.

**Row**	**Question**	**Very inappropriate**	**Inappropriate**	**Normal**	**Appropriate**	**Very convenient**
1	General environment light in work station	6	9	34	46	5
2	Topical environment light over the work desk	12	8	33	42	5
3	Light reflection	8	19	36	33	4
4	Establishment of office equipment	18	22	30	27	3
5	Position of desk relative to the window	17	27	24	32	4
6	Coloring	2	5	41	44	8
7	Desk	8	14	37	32	9
8	The chair	6	13	36	38	7
9	Work space	14	27	24	31	4
10	Location of work place compared to colleagues	18	19	31	28	4
11	Voice	37	26	19	16	2
12	Odor other than the smell of smoke	8	12	36	42	2
13	The smell of cigarettes	18	14	21	34	14
14	Dust and smoke	4	14	31	39	12
15	Humidity	6	26	32	27	9
16	The heat	16	25	27	29	3
17	The cold	16	23	25	34	2
18	Air conditioning system	22	25	26	24	3
19	For air flow	17	26	32	21	4
20	Air curtain	16	22	37	22	3
21	Average	13.4	18.8	30.6	32.05	5.35

## 2 Experimental design, materials and methods

This descriptive – cross sectional study was conducted in an office building in Mashhad in summer season of 2017. The building under study were three years old and the number of its floors with one underground floor was in total four floors and each floor had two eastern and western fronts. Floor − 1 included the place for prayer, kitchen and engine room, ground floor belonged to car parking with a capacity of about 80 car parking spaces and kitchen units, and other floors were used for administrative activities. Alberta Indoor Air Quality Toolkit was used in order to investigate indoor air pollution symptoms. The staff number for sample was selected as 80 using census. Demographic information, information related to the hours of individuals’ presence in the building and tax paid by people during presence in the building as well as their ideas regarding environmental conditions of the building were collected using the toolkit. CO_2_ was measured using TESTO direct reading system in 50 stations, CO was measured using first check in 35 stations, total particles and PM_10_ and PM_2.5_ were measured using GRIMM direct reading system in 10 stations, volatile organic compounds (VOCs) were measured using First Check at 60 stations, formaldehyde and ozone were measured using Detector Tubes in 15 stations, temperature and humidity were measured using EE07 ELECTRONIC moisture meter and E2 Read software was used for recording data in 60 stations. Smoke and sound test using sound level meter model CEL450 was used for air flow in 60 stations in the building. In each floor, a measurement was done as the criterion for respective pollutant concentration from the open air outside the building (balcony of the floor (terrace)) [13], [14], [15]. At the end, data were analyzed using Pearson statistical test in SPSS software. In order to determine frequency of staff ideas regarding health consequences of indoor air quality, scoring was done as never (1–25), rarely (26–50), sometimes (51–75), always (76–100), and following scoring method was used for investigating frequency of staff ideas on environmental factors of indoor air: highly inappropriate (1–20), inappropriate (21–40), normal (41–60), appropriate (61–80), highly appropriate (81–100).
